# Patterned Slippery Surface for Bubble Directional Transportation and Collection Fabricated via a Facile Method

**DOI:** 10.34133/2019/9139535

**Published:** 2019-11-05

**Authors:** Jian Li, Zhiguang Guo

**Affiliations:** ^1^Ministry of Education Key Laboratory for the Green Preparation and Application of Functional Materials, Hubei University, Wuhan 430062, China; ^2^State Key Laboratory of Solid Lubrication, Lanzhou Institute of Chemical Physics, Chinese Academy of Sciences, Lanzhou 730000, China

## Abstract

Directional manipulation of underwater bubbles on a solid surface has attracted much attention due to its large-scale applications such as electrocatalytic gas evolution reactions, wastewater remediation, and solar energy harvesting. In this work, the patterned slippery surface (PSS) is fabricated via a facile method where the patterned pathways are fabricated by means of etching the pristine copper sheet. These patterned surfaces consisted of pristine copper and modified oxide copper which exhibit different wettability for bubble and water. The superhydrophobic and aerophilic surface can efficiently capture bubbles, and the infused oil layer is beneficial for reducing the resistance during transportation. Furthermore, the bubble can move upward, downward, and horizontally. Hence, it is easy to realize the bubble's transportation and collection on the functional surfaces.

## 1. Introduction

The manipulation of underwater bubbles is significant in a fluid system due to its large-scale applications, such as electrocatalytic gas evolution reactions, wastewater remediation, and solar energy harvesting [1–11]. Meanwhile, the bubbles play an important role in nature. For instance, the superhydrophobic abdomen of diving bell spiders can capture bubbles for its further living in the water [12, 13]. However, the underwater bubbles also have some negative effects in industries, such as corrosion of pipes, air locks, and clogging of bubbles in intravenous tubing [14–18]. Thus, it is urgent to spend further time in research for bubble's capture and removal. In addition, there are significant correlations between liquid wettability and bubble wettability which have been reported in recent years [19–21]. The three-phase (gas-liquid-solid) contact line of bubble on a solid surface in an aqueous environment is the same as water droplet on the solid surface in air. For an ideal surface, the bubble contact angle is complementary to water contact angle. Therefore, the superhydrophobic surface presents as aerophilic while the hydrophilic surface is aerophobic. Bubbles can be captured more easily on the superhydrophobic surface than on the superhydrophilic surface.

Thus, surfaces with the super wetting ability can be applied in bubble manipulation. Tian et al. [22] have fabricated superhydrophobic PE plates with modified nanoparticles coated on it. Releasing continuous bubbles on the plate underwater, it can float and dive with bubble capture and breakage. It manifested that the superhydrophobic layer could capture bubbles efficiently, and bubbles could merge into a larger one. Yu et al. [23] realized the directional manipulation of bubbles on a superhydrophobic helix. The bubble stayed on the climax of the helix under the coeffect of buoyancy and adherence and moved directionally along the helix when it rotated. In addition, they also realized the spontaneous directional transportation of bubbles on a superhydrophobic cone [24]. In general, there is a high adhesive force between the superhydrophobic substrate and bubbles. Bubbles tend to spread rapidly and adhere to the surface due to the interaction of air and substrates [25–27]. Inspired by the movement of water droplets or insects on Pitcher plant, spider silk, and cactus spine [28–33], the slippery surfaces with a lubricant layer and the substrates with a geometrical gradient have been applied largely in the directional manipulation of underwater bubbles [7, 10, 34–41]. Zhang et al. [34] designed a wedge-shaped superhydrophobic surface with a lubricant layer via laser cutting for this directional movement. This movement occurred in a horizontal direction in which bubbles suffered unbalanced Laplace pressure, and the bubble moves entirely rather than pinned on the surface. Additionally, Tang et al. [39] used photolithograph to manufacture bioinspired patterned slippery surface which achieved upward directional transportation of buoyancy-driven bubbles. Furthermore, the directional transportation of bubbles can also be realized on the Janus membrane where bubbles can only penetrate from the hydrophilic side to the hydrophobic side [42–44]. Yin et al. [42] realized the bubble transportation through a Janus PFTE mesh which was created via a femtosecond laser. As mentioned above, the driving force for this transportation mainly depends on buoyancy and unbalanced Laplace pressure. Though there are many surfaces which are served for bubble directional transportation, the most fabricated methods are complex and time-consuming, such as laser cutting and photolithograph [24, 34, 39, 42, 43]. Thus, it is significant to explore a facile method to fabricate a functional surface for directional transportation.

Herein, a facile method has been presented in this work for fabricating the patterned slippery surfaces (PSS) for bubble manipulation. Patterns were fabricated by covering a part of the copper substrate with tape when etching. Besides, the pattern can be designed by adjusting the shape of the tape. With different shapes on the slippery surface, the manipulations of bubble can be controlled. Furthermore, bubbles can move upward with different paths by buoyancy, horizontal and downward on PSS with geometrical gradient under unbalanced Laplace pressure. This facile method is beneficial for further study of bubble manipulation.

## 2. Result and Discussion

### 2.1. Preparation of Patterned Slippery Surface and Characterization

**Figure 1:**
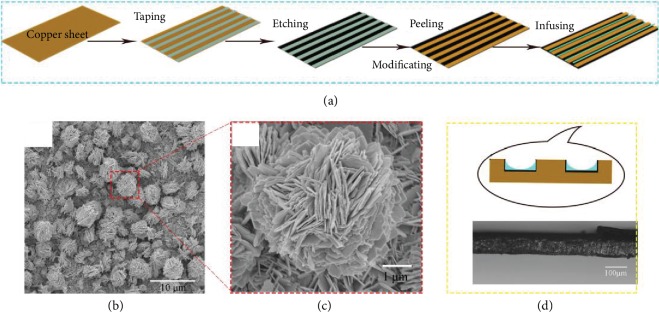
(a) Schematic of the fabricated process of PSS sample. (b) The SEM images of oxide copper. (c) The magnified SEM images of oxide copper in (b). (d) Schematic and optical image of the side view of PSS-1.

**Figure 2:**
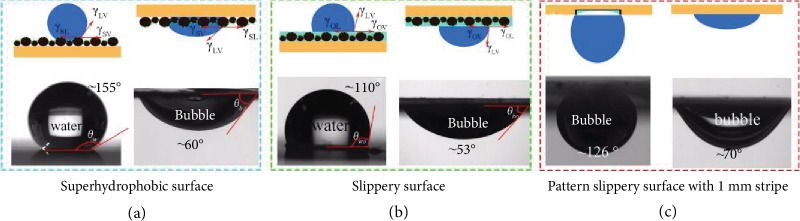
Wetting states of water and bubbles on different substrates. (a, b) Water droplet and bubble on superhydrophobic surface and slippery surface, respectively. (c) Bubble on PSS-1.

The etching process was expressed as previous work [45]. The whole fabrication process of this patterned slippery surface was shown in Figure 1. The transparent tape was functional for the protection to avoid pristine copper to be etched which resulted in a patterned surface, the yellow part reflected the pristine copper, and the black part meant the oxide copper after being etched. Figures 1(a)–1(c) show the detailed morphologies of the modified oxide copper and the section view of the patterned surface. The oxide copper showed a spherical shape, and the section view displayed a concave-convex shape. The oxide copper was in the lower part. Besides, the width ratio between the yellow and black parts is 1 : 1. Furthermore, the images of bubble and water contact angles on different types of surfaces (superhydrophobic surfaces, slippery surfaces, original surfaces, and PSS) were shown in Figure 2, [Supplementary-material supplementary-material-1], and [Supplementary-material supplementary-material-1]. In addition, the black part was modified to be superhydrophobic and aerophilic with the water and bubble contact angles of 155 ± 2° and 60 ± 2°; the pristine copper is hydrophilic and aerophobic of water and bubble contact angles of 80 ± 2° and 130 ± 2°. For the ideal surface, the water contact angle (*θ*_*w*_) in air and the bubble contact angle (*θ*_*b*_) in water complementary resulted from almost the same three-phase contact line. Additionally, the effect of buoyancy for bubble was analogous of gravity for water droplet. According to Young's equation, the values of *θ*_*w*_ and *θ*_*b*_ can be calculated as

(1) $$\begin{array}{c} \cos\theta_{w}=\frac{\left(\gamma_{\text{SV}}-\gamma_{\text{SL}}\right)}{\gamma_{\text{LV}}}, \end{array}$$

(2) $$\begin{array}{c} \cos\theta_{b}=\frac{\left(\gamma_{\text{SL}}-\gamma_{\text{SV}}\right)}{\gamma_{\text{LV}}}, \end{array}$$

where *γ*_SV_, *γ*_LV_, and *γ*_SL_ represented the surface energy of solid-vapor interface, liquid-vapor interface, and solid-liquid interface, respectively. Based on equation (1) and (2), it is easy to derive *θ*_*b*_ = 180° − *θ*_*w*_. After lubricant PFPE was infused, the oil layer had substituted the solid substrate which resulted in the change of three phases. Therefore, the water (*θ_wo_*) and bubble (*θ_bo_*) contact angles were 110 ± 2° and 53 ± 2° with oil layer, which can be expressed as

(3) $$\begin{array}{c} \cos\theta_{wo}=\frac{\left(\gamma_{\text{OV}}-\gamma_{\text{OL}}\right)}{\gamma_{\text{LV}}},\\ \cos\theta_{bo}=\frac{\left(\gamma_{\text{OL}}-\gamma_{\text{OV}}\right)}{\gamma_{\text{LV}}}, \end{array}$$

where *γ*_OV_ and *γ*_OL_ are the surface energy of oil-vapor interface and oil-liquid phase. Besides, *θ_wo_* and *θ_wb_* were still complementary, *θ*_*wo*_ + *θ*_*wb*_ = 180°. In addition, the patterned surface displays anisotropic wettability that resulted from the wettability difference between the yellow and black parts; the bubble contact angle is 126 ± 2° and 70 ± 2° in the *x* and *y* directions on the treated part with 1 mm width. From these results, it can be concluded that the treated part remains aerophilic for better bubble capture and transportation.

### 2.2. Directional Manipulation of Bubble on PSS with Straight Stripes

**Figure 3:**
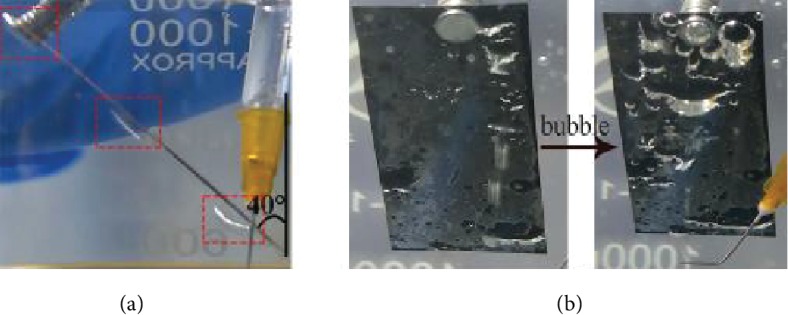
Bubble movement on different substrates. (a) Pristine copper. (b) Modified oxide copper.

As for the whole process of bubble directional manipulation, the first step is capturing the bubbles. As shown in Figure 3(a), the pristine copper sheet cannot capture bubbles. Bubbles bounced to the upper position continuously to escape after it contacted with the inclined samples since the pristine copper is aerophobic. Moreover, the directional manipulation of underwater bubbles cannot be realized on the superhydrophobic oxide copper. From Figure 3(b), the silver mirror-like phenomenon was observed immediately on the superhydrophobic sample after being immersed in water; there was a bubble layer on the sample. Additionally, although the substrate can capture bubbles efficiently, bubbles stay in the original position to expand the air layer instead of moving upward, which was shown in Figure 3(b). Thus, it is hard to realize the directional transportation of underwater bubbles on superhydrophobic substrates, while in previous work the directional transportation of bubbles has been accomplished by a slippery surface with shape gradient structure [25].

**Figure 4:**
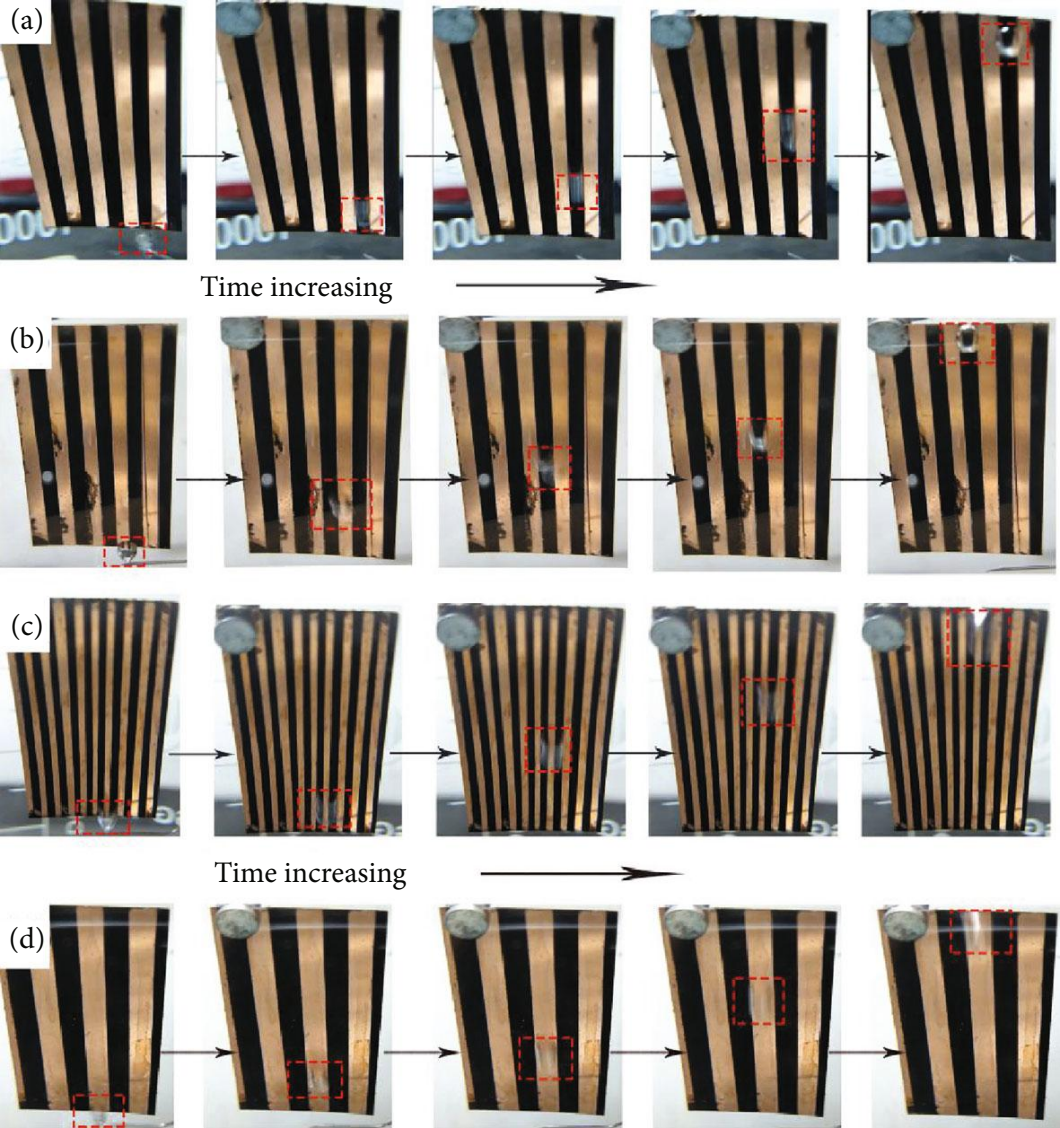
The directional transportation of single bubble with the volume about 15 *μ*L on the PSS-2 with different initial position: (a) treated stripe and (b) untreated stripe. The special transportation of bubble on different PSS samples. (c) Bubble covered three stripes during the transportation in PSS-1. (d) Bubble moved on the untreated stripe in PSS-3.

**Figure 5:**
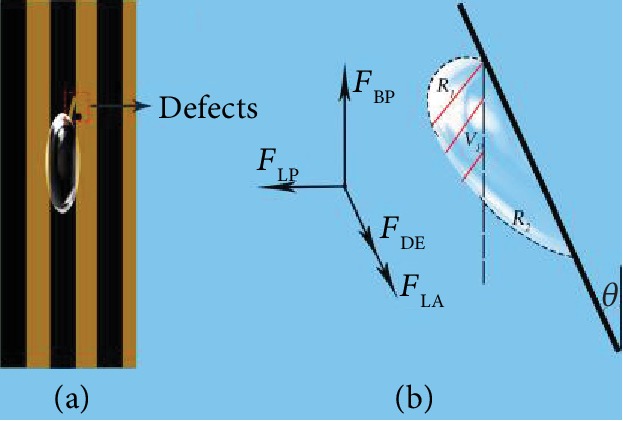
Schematic of bubble shape on the treated stripe in top view (a) and side view (b) and the force condition for the bubble.

Therefore, based on those researches, the slippery surface fabricated by the superhydrophobic substrate with infused oil layer was conducive to capture and transport bubbles [25, 34–37, 39]. In this work, we have fabricated patterned slippery surface (PSS) with different widths to achieve the goal of directional manipulation. As shown in Figure 4 and [Supplementary-material supplementary-material-1], the PSS-2 was inclined in water to observe the bubble movement. Then, releasing a single bubble with the volume of about 15 *μ*L in the slippery stripe, it was restricted by the boundary on the treated stripes and moved from the bottom up with the shape of ellipse (Figure 4(a)). Besides, releasing a single bubble with the same volume in the untreated stripe, the bubble bounced on the stripe to the upper location firstly, then it moved horizontally to the slippery area that resulted from the touch between the bubble and the slippery stripe. Then, the bubble transported to the top on the slippery stripe after horizontal movement (Figure 4(b)). From the single bubble motion on the PSS-2, it can be concluded that the combined surface can realize the directional manipulation of underwater bubbles on the treated stripes. Further, the same phenomenon had been observed in samples of PSS-1 and PSS-3 as shown in Fig. [Supplementary-material supplementary-material-1] and [Supplementary-material supplementary-material-1] with the same volume of bubble. Bubbles appeared in different shapes in treated stripes with different widths that resulted from the tendency that bubble would cover more area on the treated stripes. The transportations were also shown in [Supplementary-material supplementary-material-1] and [Supplementary-material supplementary-material-1]. However, there were some special phenomena in the directional manipulation in the samples PSS-1 and PSS-3 with the single bubble with the volume of 15 *μ*L. As shown in Figure 4(c), the bubble covered three stripes (treated, untreated, and treated) during the whole upward transportation with the first contacted position of the untreated stripe, while in the sample of PSS-3 the bubble moved from the bottom up in the untreated part instead of a horizontal movement to the treated stripe to the top, which was shown in Figure 4(d). The special transportations were also shown in [Supplementary-material supplementary-material-1]. It can be ascribed to the relationship of bubble volume and stripe width. In PSS-1, the bubble tended to move horizontally to the treated part after being released on the untreated part, but the stripe width was much narrower compared to the bubble diameter that resulted in the touch between the bubble and two treated stripes. Then, the bubble and three stripes achieve a balance for directional movement. Nevertheless, the sample width of PSS-3 was too large for this bubble which resulted in the bouncing of the bubble from bottom up on the untreated stripe. From those movements of 15 *μ*L bubble on the PSS samples, it could be concluded that the directional movement was mainly decided by the treated stripe which offered the capacity of capturing bubbles and the transport area. Furthermore, the horizontal movement depended on the contact of the bubble and the treated part. The treated part is also aerophilic with the oil layer for bubbles containing lower free energy than the pristine copper part that resulted in better capturing of the bubbles. Moreover, the added oil layer can make the substrate more regular and polisher to cover defects of this substrate. Based on this oil layer, bubbles can move entirely instead of bulk of movement with part of it remaining in the initial position under the driving force of buoyancy. In addition, the driving force for the bubble directional transportation included buoyancy (*F*_BP_) and Laplace force difference (*F*_LP_), and the resistance was mainly generated by the adhesive force (*F*_AD_) and the sample defects (*F*_DE_), which was shown in Figure 5. Furthermore, the buoyancy was generated by a part of the bubble which was the unbounded region, *F*_BP_ can be calculated as [46, 47]

(4) $$\begin{array}{c} F_{\text{BP}}=\rho gV_{P}, \end{array}$$

where *ρ* is the density of water, *g* is the gravitational acceleration, and *V_P_* is the effective volume of the bubble (the left part of bubble in Figure 5(b)). Moreover, there is a Laplace force difference (*F*_LP_) generated by the curved bubble surface; it can be calculated as [47]

(5) $$\begin{array}{c} F_{\text{LP}}=\frac{2\gamma_{\text{water}}}{R_{1}}A_{1}-\frac{2\gamma_{\text{water}}}{R_{2}}A_{2}, \end{array}$$

where *γ*_water_ is the surface tension of water, *R*_1_ and *R*_2_ are the radii of bubble in the front and back sides, and *A*_1_ and *A*_2_ are the projected area of the bubble at the front and back sides. In addition, in those inclined samples, the adhesion belonged to the lateral adhesion (*F*_LA_) which its direction is against the directional transportation of bubbles on a solid substrate [46–49]. Thus, the resultant force (*F*_RT_) in the moving direction can be calculated as

(6) $$\begin{array}{c} F_{\text{RT}}=F_{\text{BP}}\cos\theta +F_{\text{LP}}\sin\theta -F_{\text{LA}}-F_{\text{DE}}, \end{array}$$

where *θ* is the inclined angle of the surface in water. Based on equation (6), the movement was affected by several factors. *F*_BP_ and *F*_LP_ mainly depended on the inclined angle; *F*_LA_ had been reduced by the oil layer that resulted in the directional transportation. From above experiments, it can be confirmed that the PSS samples with straight treated stripes can realize efficiently the directional transportation of bubbles. Further, the samples also reduced the modified area in the total surface but with the same capacity of capturing bubbles compared to the whole modified surface which was beneficial for the environment. Hence, the patterned surface with the oil layer is an ideal candidate for capturing and transporting bubbles underwater.

### 2.3. Directional Transportation of Bubbles on PSS Samples with Special Shapes

**Figure 6:**
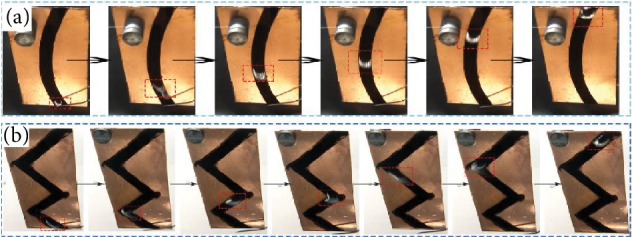
The images of bubble directional transportation on different designed samples: (a) PSS-C and (b) PSS-M.

**Figure 7:**
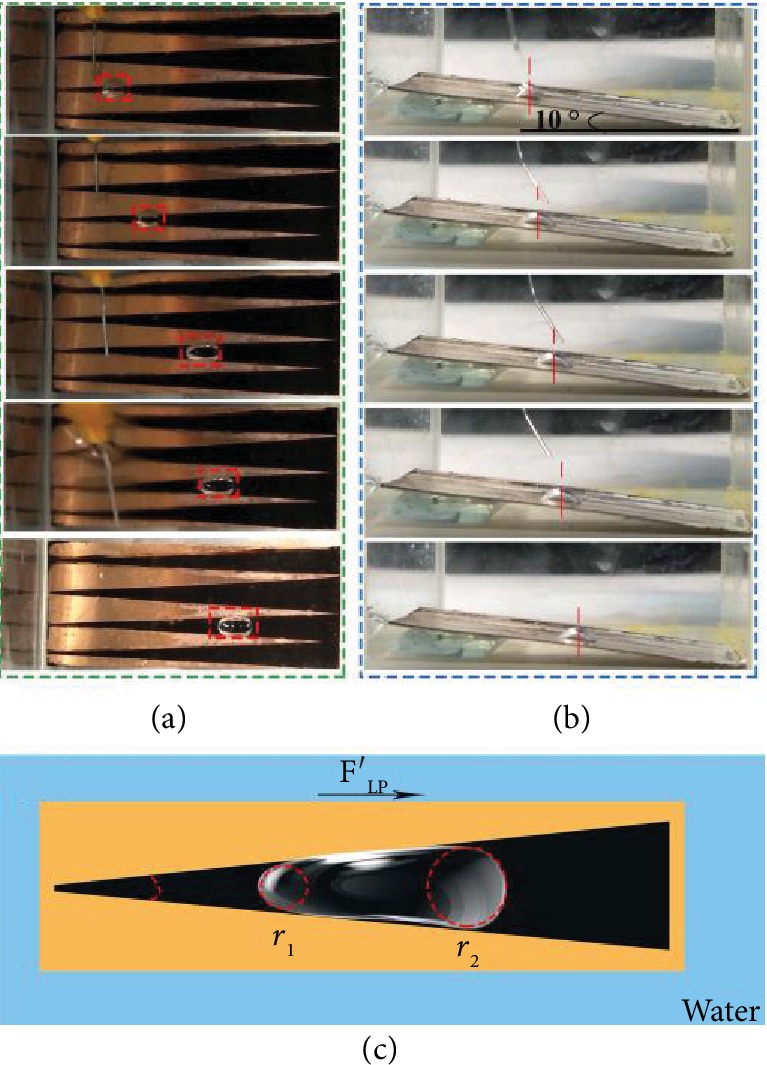
The images of bubble on the PSS-wedge of the directional transportation: (a) horizontal and (b) inclined. (c) The mechanism of bubble transport on the PSS-wedge.

Based on the fabricated method of the above samples with straight pathway, we also had fabricated the patterned slippery surface with the treated part displayed letter “C” (PSS-C). The width of the treated path was 4 mm. As shown in Figure 6(a), the bubble with a volume of 15 *μ*L moved from the bottom up in a curve track which was the same as the treated path. The boundary line had played an important role in preventing the bubble escape from the treated path. Thus, the treated path on PSS samples offered the transported tracks for bubbles after bubble releasing. We also fabricated the patterned slippery surface with the treated part displayed letter “M” (PSS-M). There were some turning points in this sample. Therefore, the single bubble with the volume of about 15 *μ*L was released on the PSS-M to detect the ability of directional transportation on this sample, which was shown in Figure 6(b) and [Supplementary-material supplementary-material-1]. The width of “M” was 3 mm, and the boundary lines were parallel and straight. The single bubble displayed a circular shape and moved along the treated path. Then, the bubble turned right and left when it reached the turning point of letter “M” in order to remain on the treated part all the time. Moreover, the shape of the bubble changed largely when going through the turning point. The transported track was also the same as the treated path, the letter “M.” With the letters “M” and “C” on the substrate, we can control the directional transportation of bubbles underwater more precisely in ideal directions and paths. Although those PSS samples (PSS-1, PSS-2, PSS-3, PSS-C, and PSS-M) could realize the directional transportations, bubbles could only move vertically from the lower position to the higher position driven by buoyancy force. Nonetheless, this facile method of fabricating the PSS samples offered the opportunity for spontaneous directional transportation of bubbles underwater. Thus, we fabricated the PSS samples with wedge shapes for spontaneous directional transportation of bubbles. There were four equal wedges on the copper sheet with the apex angle of 7°. As shown in Figure 7(a) and [Supplementary-material supplementary-material-1], the bubble moved from the narrow tip to the wide end after being released on the wedge spontaneously. Besides, the bubble moved faster in the early time and slowed down to stagnation. Moreover, this PSS-wedge sample could also realize antibuoyancy movement. As shown in Figure 7(b) and [Supplementary-material supplementary-material-1], the bubble moved downward spontaneously after being released on the inclined sample. The driving force on this sample of the PSS-wedge was the Laplace force difference (*F*_LP_′) no matter how the bubble moved. As shown in Figure 7(c), *F*_LP_′ can be calculated as [34, 50]

(7) $$\begin{array}{c} F\prime_{\text{LP}}=\left(\gamma_{\text{water}}-\gamma_{\text{oil}}\right)\left(\frac{1}{r_{1}}-\frac{1}{r_{2}}\right)\frac{\sin\alpha }{r_{2}-r_{1}}V, \end{array}$$

where *γ*_oil_ is the surface tension of the oil layer, *α* is the apex angle, *V* is the bubble volume, and *r*_1_ and *r*_2_ are the radii of the bubble in the tip and end positions. For the movement in Figure 7(b), the buoyancy became the resistance. Thus, the transported distance was much shorter than that in the inclined PSS-wedge sample. Hence, the patterned slippery surface can be designed with serval special shapes on it by covering the pristine copper sheet for further directional transportation of bubbles.

### 2.4. Bubble Transportation and Collection on PSS-Y

**Figure 8:**
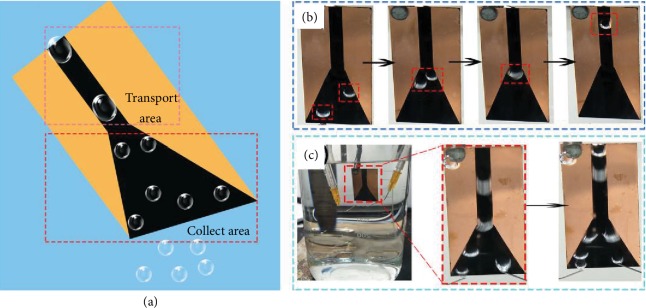
The movement of bubbles on PSS-Y. (a) Schematic of this directional movement and collection. (b) The movement of two bubbles on PSS-Y. (c) The bubble collection on this sample.

Bubble capture and collection have attracted much attention in recent years. Though there were many materials investigated for bubble collection, simplifying the fabrication methods for those materials was still a challenge. However, the PSS samples were fabricated via a facile etching method and it could efficiently capture bubbles and transport them along the treated area. Therefore, we designed a sample (PSS-Y) based on this method to achieve the goal of bubble collection. This sample also included two parts, the treated and untreated parts. As shown in Figure 8(a), the treated part was combined by two areas, the collect area and the transport area. Moreover, the transport area was 4 mm wide and 20 mm long which showed a rectangle, while another area showed a trapezoid with the upper and lower widths of 4 mm and 20 mm, respectively, and the height was 20 mm. The total process of bubble collection could be divided into three steps: attach on the collect area, move on to the transport area, and escape from this sample. We first examined the movement of two individual bubbles on this PSS-Y sample. As shown in Figure 8(b) and [Supplementary-material supplementary-material-1], two bubbles with the volume of about 15 *μ*L moved upward along the boundary line in the lower part. Then, the two bubbles merged into one when they moved to the joint between the straight and lower part. Finally, the merged bubble transported on the stripe to the end along the treated straight path. From the transportation of bubbles on the sample of PSS-Y, this special PSS samples could collect bubbles by the two parts. Based on the phenomenon of two bubbles on PSS-Y, we have released multiple bubbles on PSS-Y in different positions. From Figure 8(c) and [Supplementary-material supplementary-material-1], all bubbles were captured by the collect area and moved along the treated part. Furthermore, bubbles might coalesce in the joint and move upward to escape this sample in the straight treated path. In this sample of PSS-Y, the trapezoid part had amplified the contact area between bubbles and sample which resulted in larger collect area, and the straight part restricted the leaving position of bubbles for bubble collection.

## 3. Conclusion

In summary, the directional transportation and collection of bubbles underwater were realized on the patterned slippery surfaces which were fabricated via a facile method. These samples of PSS were combined by two regions, the treated superhydrophobic part with lubricant infused and the untreated part of the pristine copper. The superhydrophobic and aerophilic surface can efficiently capture bubbles, and the infused oil layer was beneficial for reducing the resistance for bubble transportation. Hence, the samples were capable of capturing and transporting bubbles on the treated part. Further, with a well-designed path on the surface such as PSS-C, PSS-M, and PSS-wedge, the directional transportation of bubbles can be organized precisely. Furthermore, the PSS-Y can realize the bubble's collection. This facile method demonstrated promising prospective in fabricating substrates for directional transportation and collection of bubbles.

## 4. Experimental Section

### 4.1. Materials

All reagents are obtained as listed. Pentadecafluorooctanoic acid (PFOA) was obtained from Saen Chemical Technology Co., Ltd.; hydrochloric acid (HCl) was obtained from Xilong Scientific. Ammonium persulfate (98%, (NH_4_)_2_S_2_O_8_), sodium hydroxide (96%, NaOH), ethyl alcohol, and acetone were purchased from Tianjin Rionlon Pharmaceutical Science & Technology Development Co., Ltd. Copper foils were supplied by Sinopharm Chemical Reagent Co., Ltd. The transparent tape was purchased from a local supermarket. The DuPont Krytox perfluoroalkylpolyether (PFPE, >95.0%) GPL 103 lubricant was purchased from the Chemours Company. The bubbles were generated by the syringe of air bubble. Deionized (DI) water was purified using a ModuPure system and used throughout the study.

### 4.2. Preparation of Patterned Slippery Surface

Copper sheet (20 mm × 40 mm) was cleaned several times by acetone, ethanol, dilute hydrochloric acid, and deionized water under consecutive ultrasonication for about 40 minutes. Put the tapes (1 mm × 40 mm, 2 mm × 40 mm, and 3 mm × 40 mm) on the substrate line by line with the spacing of 1 mm, 2 mm, or 3 mm after drying the substrate in an oven at 60°C. Then, put the samples into a mixed etching solution of 100 mL (2 mol/L NaOH, 0.1 mol/L (NH_4_)_2_S_2_O_8_) for 3 h to obtain CuO on the uncovered surface. After that, peel the tape to obtain the patterned surface which was combined by pristine copper and etched copper. Dry the surface by N_2_ and modify it by PFOA water solution (0.01 mol/L) to obtain superhydrophobicity of the treated part. At last, an amount of PFPE was applied on the patterned surfaces to realize the patterned slippery surfaces with different stripe widths (PSS-1, PSS-2, and PSS-3).

### 4.3. Characterization

Field emission scanning electron microscope (FESEM, JSM-6701F) with Au-sputtered specimens was served to obtain detailed morphologies of the surface. The contact angles of water and bubble on serval samples were measured by a JC2000D goniometer (Zhongchen Digital Equipment Co. Ltd., Shanghai, China). All photographs and videos were captured by a mobile phone and a digital camera (Sony camera, DSCHX200). The average contact angles were measured in the same sample in five different positions.

### 4.4. Directional Manipulation and Collection of Bubbles

Those bubble manipulations included two types, the buoyancy-driven and the self-driven. Moreover, the positions of those samples were different. The samples for buoyancy-driven were fixed underwater vertically with an inclined angle of 40°, while the others were horizontally placed in the container. Bubbles were generated by the syringes.

## Supplementary Materials

Supplementary 1 Table S1: the values of water and bubble contact angles in different wetting states. Figure 1: images of water or bubble contact angles on various surfaces: (a) pristine copper, (b, c) PSS-2, and (d) PSS-3. Figure 2: directional transportation of a single bubble on PSS-1 with different initial contact position: (a) the treated stripe and (b) the untreated stripe. Figure 3: directional transportation of a single bubble on PSS-3 with different initial contact position: (a) the treated stripe and (b) the untreated stripe.

Supplementary 2 Movie 1: directional transportation of a single bubble on PSS 1, 2, and 3.

Supplementary 3 Movie 2: directional transportation of a single bubble on PSS with special shapes.

Supplementary 4 Movie 3: bubble movement on PSS-Y.
