# Supplementary material for: Patterned Slippery Surface for Bubble Directional Transportation and Collection Fabricated via a Facile Method
Source: Research (Wash D C). 2019 Nov 5;2019:9139535. doi: 10.34133/2019/9139535 (PMC6946277; doi:10.34133/2019/9139535)
Supplement: Supplementary 1 — Table S1: the values of water and bubble contact angles in different wetting states. Figure 1: images of water or bubble contact angles on various surfaces: (a) pristine copper, (b, c) PSS-2, and (d) PSS-3. Figure 2: directional transportation of a single bubble on PSS-1 with different initial contact position: (a) the treated stripe and (b) the untreated stripe. Figure 3: directional transportation of a single bubble on PSS-3 with different initial contact position: (a) the treated stripe and (b) the untreated stripe. [file 9139535.f1.docx]

**Support information**

**Pattern slippery surface for directional bubbles transport and collection fabricated by a facile method**

*Jian Li ^a, b^ and Zhiguang Guo ^a, b*^*

a. Ministry of Education Key Laboratory for the Green Preparation and Application of Functional Materials, Hubei University, Wuhan 430062, People’s Republic of China

b. State Key Laboratory of Solid Lubrication, Lanzhou Institute of Chemical Physics, Chinese Academy of Sciences, Lanzhou 730000, People’s Republic of China.

*Corresponding author. Tel: 0086-931-4968105; Fax: 0086-931-8277088. Email address: zguo@licp.cas.cn (Guo)

Table.S1 Wettability of various surfaces

| Samples | | P-Cu | S-CuO | Oil-CuO | PSS-1 | PSS-2 | PSS-3 |
| --- | --- | --- | --- | --- | --- | --- | --- |
| Water  contact  angles (°) | | 80±2 | 155±2 | 110±2 |  |  |  |
| Bubble  contact  angles (°) | *X* | 130±2 | 70±2 | 53±2 | 126±2 | 110±2 | 95±2 |
|  | *Y* |  |  |  | 70±2 | 66±2 | 54±2 |

P-Cu: Pristine copper; S-Cu: Superhydrophobic oxide copper; Oil-CuO: PFPE infused sample. X means the direction parallel the treated path, Y means the vertical direction.


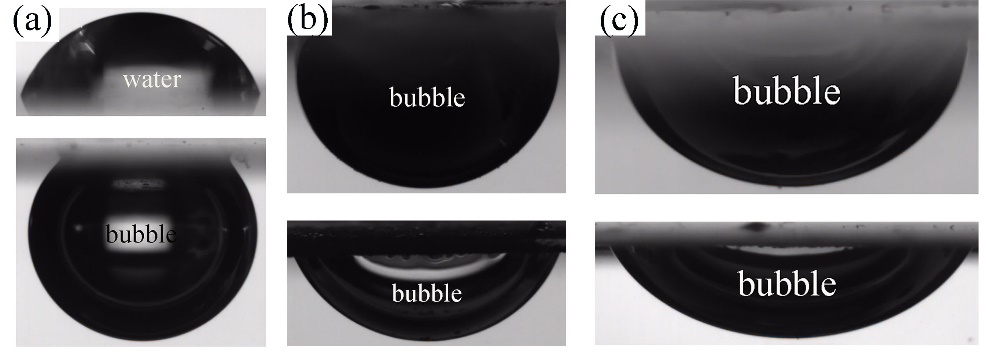


Fig.S1 Images of water or bubble contact angles on various surfaces. (a) pristine copper, (b) (c) PSS-2, (d) PSS-3.


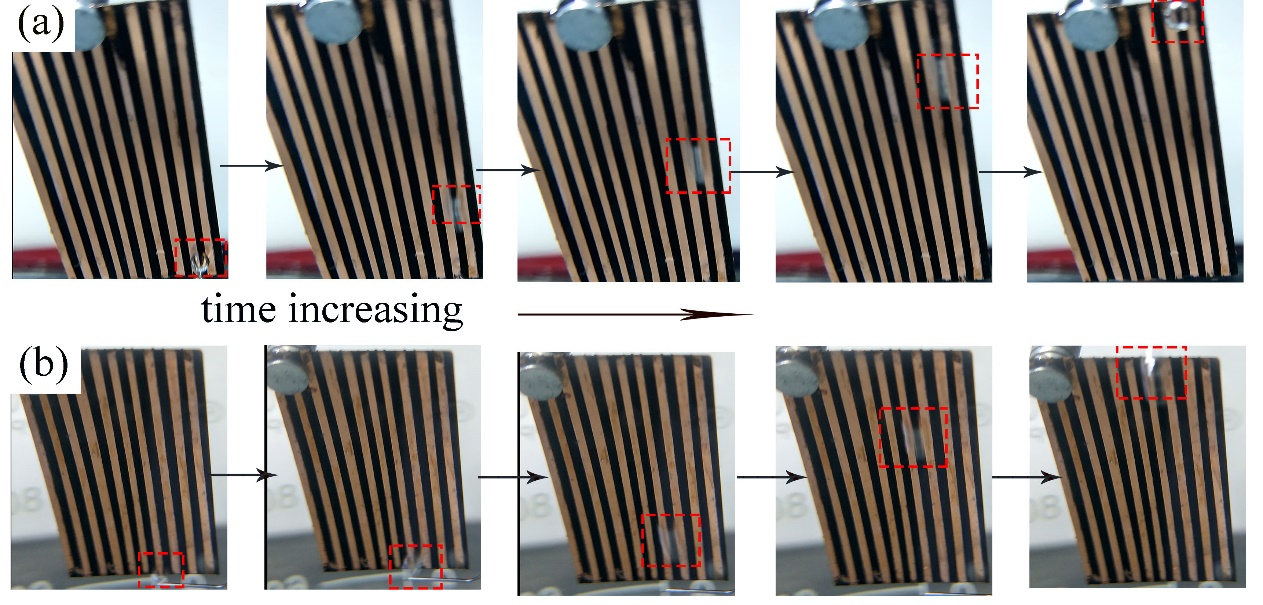


Fig. S2 Directional transportation of single bubble on PSS-1 with different initial contact position,(a) the treated stripe, (b) the untreated stripe.


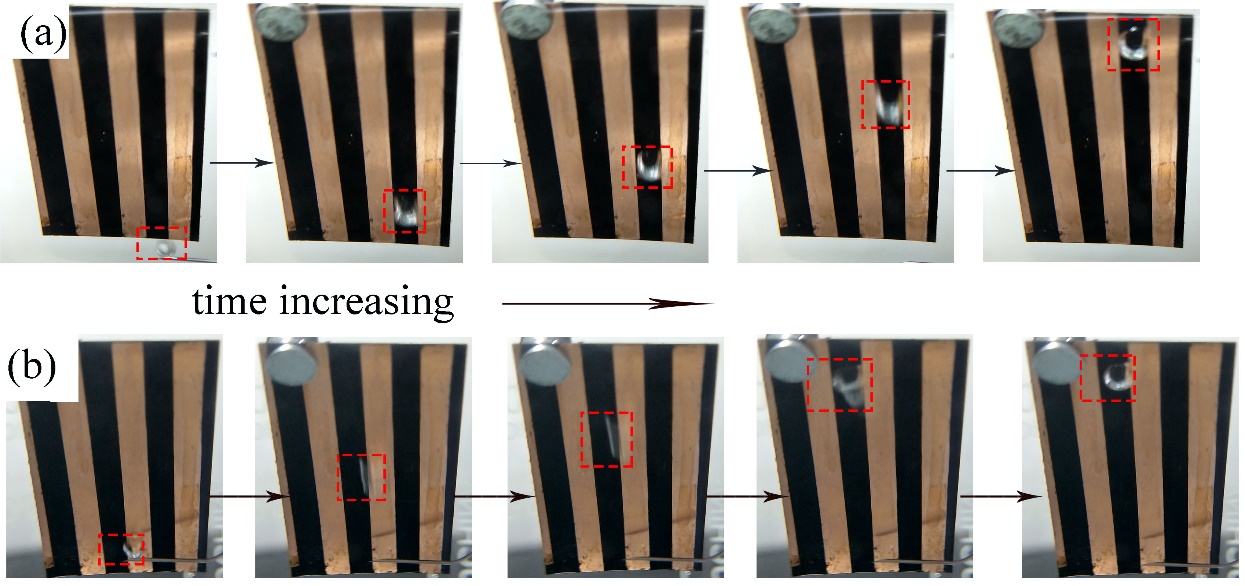


Fig. S3 Directional transportation of single bubble on PSS-3 with different initial contact position,(a) the treated stripe, (b) the untreated stripe.
